# Lipid raft-based membrane order is important for antigen-specific clonal expansion of CD4^+^ T lymphocytes

**DOI:** 10.1186/s12865-014-0058-8

**Published:** 2014-12-14

**Authors:** Daniel Schieffer, Sanya Naware, Walter Bakun, Anil K Bamezai

**Affiliations:** Department of Biology, Villanova University, 800 Lancaster Avenue, Villanova, PA 19085 USA; Current Address: DeNovix Inc, Wilmington, DE 19808 USA; Current Address: M.D. Program, Drexel University, 3141 Chestnut Street, Philadelphia, PA 19104 USA

**Keywords:** Lipid rafts, Membrane order, CD4^+^ T cells, Clonal expansion, Cholesterol, 7-ketocholesterol, Fluorescence resonance energy transfer

## Abstract

**Background:**

Lipid rafts are cholesterol and saturated lipid-rich, nanometer sized membrane domains that are hypothesized to play an important role in compartmentalization and spatiotemporal regulation of cellular signaling. Lipid rafts contribute to the plasma membrane order and to its spatial asymmetry, as well. The raft nanodomains on the surface of CD4^+^ T lymphocytes coalesce during their interaction with antigen presenting cells (APCs). Sensing of foreign antigen by the antigen receptor on CD4^+^ T cells occurs during these cell-cell interactions. In response to foreign antigen the CD4^+^ T cells proliferate, allowing the expansion of few antigen-specific primary CD4^+^ T cell clones. Proliferating CD4^+^ T cells specialize in their function by undergoing differentiation into appropriate effectors tailored to mount an effective adaptive immune response against the invading pathogen.

**Results:**

To investigate the role of lipid raft-based membrane order in the clonal expansion phase of primary CD4^+^ T cells, we have disrupted membrane order by incorporating an oxysterol, 7-ketocholesterol (7-KC), into the plasma membrane of primary CD4^+^ T cells expressing a T cell receptor specific to chicken ovalbumin_323–339_ peptide sequence and tested their antigen-specific response. We report that 7-KC, at concentrations that disrupt lipid rafts, significantly diminish the c-Ovalbumin_323–339_ peptide-specific clonal expansion of primary CD4^+^ T cells.

**Conclusions:**

Our findings suggest that lipid raft-based membrane order is important for clonal expansion of CD4^+^ T cells in response to a model peptide.

**Electronic supplementary material:**

The online version of this article (doi:10.1186/s12865-014-0058-8) contains supplementary material, which is available to authorized users.

## Background

Spatial distribution of signaling molecules/receptors within the plasma membrane and their re-organization during cellular interaction appears to be important for responses generated by immune and non-immune cells [1-7]. While asymmetry in the plasma membrane is intrinsic because of the distribution of lipids that harbor either positive or negative charge [8-12], the compositionally heterogeneous lipid rafts [13-19] contribute to membrane asymmetry, as well. Lipid rafts are enriched in saturated lipids, lipid-anchored proteins including ones with glycosylphosphatidyl-linkage, and cholesterol [20-24]. The distribution of cholesterol in the membrane and compositional heterogeneity of lipid rafts generates lipid raft-dependent membrane order and spatial asymmetry on the plasma membrane. Ways to disrupt lipid raft-based membrane order and molecular asymmetry in the membrane and assess its consequence on cellular responses have not been fully tested.

CD4^+^ T cells play a central role in orchestrating the adaptive immune response in vertebrates. The antigen receptor on CD4^+^ T cells recognizes a specific antigen being displayed via the Major Histocompatibility Complex (MHC) on the surface of antigen presenting cells (APC) [25,26]. A number of other accessory cell proteins with co-stimulatory function provide additive or synergistic signaling [27]. All these signaling proteins congregate at the contact site of the two interacting cells and form an immunological synapse [28,29]. Lipid rafts with their cargo are recruited to this site [30-35]. These early membrane events unleash signaling cascades that result in activation of three key transcriptional factors, namely NFAT, NFkB, and AP-1, which in turn drive transcription of, among others, the gene for T cell growth factor, IL-2. T cell growth factor-dependent clonal expansion of CD4^+^ T cells is key to the cell-mediated adaptive immune response to a foreign antigen. It is during this phase that the CD4^+^ T cells differentiate in response to intrinsic (cell-autonomous) and extrinsic (non-cell autonomous signaling initiated by cytokines derived from cells of innate immunity) factors into Th1, Th2, Th17 or T_reg_ effector T cells for generating effective immunity against invading pathogens.

A number of signaling receptors, ion channels and cell signaling proteins are sequestered in lipid rafts [36-40], but the role of these cholesterol-rich nanodomains in CD4^+^ T cell signaling has remained unclear. One mechanism through which lipid rafts may contribute to cell signaling in CD4^+^ T cells is by promoting dynamic asymmetry in the plasma membrane and allowing interactions between signaling proteins as the sub-populations of nano-domains, each housing signaling proteins, coalesce [2,41]. Recently we have observed that the initial contact between the CD4^+^ T cell and the APC, in the absence of a specific antigen, promotes lipid raft coalescence [42]. However, the role of lipid raft-based membrane order in clonal expansion of primary CD4^+^ T cells in response to a specific foreign antigen is not fully examined.

One approach to assess the role of lipid raft-based order in cell signaling is by disrupting the membrane order, either by removing cholesterol from these nano-domains or inserting raft-destabilizing molecules in them. MβCD, a compound that binds cholesterol and destabilizes lipid rafts, and has been used to assess the role of lipid rafts during the early phase of cell signaling [43,44]. However, the effectiveness of this compound at high concentrations over a short incubation period (15 min) and its adverse effects on internal Ca^2+^ stores has raised concerns over its use [45-47]. Therefore to test the role of lipid raft-based order in CD4^+^ T cell response, we have inserted a naturally occurring oxysterol, 7-KC, into the plasma membrane of CD4^+^ T cells to disrupt the lipid raft-dependent order. Incorporation of 7-KC with its ketone group at the 7^th^ position of the sterol ring disrupts the liquid ordered (I_o_) phase of model membranes and promotes formation of liquid disordered (I_d_) phase [41]. We have examined the role of lipid raft-based membrane order in clonal expansion of CD4^+^ T cells after inserting 7-KC into the membrane and disrupting the membrane order. Our results show that disruption of lipid raft-dependent membrane order with incorporation of 7-KC in the membrane negatively impacts antigen-specific clonal expansion of primary CD4^+^ T cells.

## Methods

### Mice

We used DO.11 TCRαβ transgenic (a generous gift from Dr. Dennis Loh) [48] and Balb/c mice. Mice were housed at Villanova University vivarium with active sentinel program in accordance with approved internal IACUC protocols and guidelines. Research involving mice reported in the manuscript was approved by institutional IACUC committee as per guidelines set up by the Office of Laboratory Animal Welfare (OLAW) at National Institutes of Health.

### Purification of CD4^+^ T cells

Lymph nodes were dissected from mice and single cell suspension was generated by gently crushing them between frosted glass slides and harvesting in wash medium made with RPMI 1640 supplemented with 5% fetal bovine and 2 mM HEPES buffer (Invitrogen-Life Technologies, Grand Island, NY). CD4^+^ T cells were negatively selected from total LN cell preparations using magnetic beads according to the manufacturer’s protocol (Invitrogen-Life Technologies, Grand Island, NY).

### Antigen presenting cells (APCs)

Autologous spleen cells from Balb/c mice were used as a source of APCs. A single cell suspension generated from spleen was treated with Tris-ammonium chloride for 7 minutes at 37°C to lyse red blood cells. Spleen cells devoid of RBC were washed with wash media (RPMI 1640 supplemented with 5% fetal bovine serum and 2 mM HEPES) and re-suspended in culture media with cOva_323–339_ peptide for presentation to CD4^+^ T cells.

### Cell culture

Lymph node cells or purified DO.11 CD4^+^ T cells were cultured in the presence or absence of cOva_323–339_ peptide (Sigma Genosys, Woodlands, TX). In some co-cultures syngeneic APC’s from Balb/c mice were included. To assess the proliferation of CD4^+^ T cells, lymph node cells or purified CD4^+^ T cells were labeled with CFSE (Molecular Probes-Life Technologies, Grand Island, NY) prior to their addition to the co-cultures as per previously published protocols with some modification [49]. Briefly, CFSE staining was carried out with 5×10^6^ cells at 600 nM final concentration in 1 ml volume for 10 minutes, with gentle shaking at ambient temperature. The staining was quenched by adding two volumes of ice cold wash media followed by incubation on ice for five minutes. The cells were pelleted by centrifugation and the washing step was repeated twice to remove excess CFSE dye. Cells were then re-suspended in culture media consisted of RPMI 1640 supplemented with 10% FBS, 2 mM of non-essential amino acids, 50 I.U/ml penicillin and 50 μg/ml streptomycin and 1 μg/ml fungizone, 2 mM HEPES (Invitrogen-Life Sciences, Grand Island, NY). 5x10^5^ CFSE labeled lymph node cells or 1 × 10^5^ CFSE-labeled CD4^+^ T cells were cultured with either the control cOva_324–334_ or stimulatory cOva_323–339_ peptide at 1 μM final concentration of the peptide for 72 hrs at 37°C in a 5% CO_2_ incubator. For cultures with purified CD4^+^ T cells, 2.5×10^5^ syngeneic APC were added to present specific peptide. Additional co-cultures were set up with CD4^+^ T cells that were not labeled with CFSE to serve as a control for analysis by flow cytometer.

### Treatment of cells with 7-keto cholesterol (7-KC) and methyl β cyclo-dextrin (MβCD)

7-KC and MβCD complexes were generated and incorporated into the plasma membrane by following a previously published protocol [50]. Briefly, cells were treated with a mixture of an appropriate concentration of 7-KC (Sigma-Aldrich, St-Louis, MO) ranging from 70 μM to 17.5 μM and a fixed concentration (0.3 mM) of MβCD (Sigma-Aldrich, St-Louis, MO). 7-KC-MβCD complexes were added to the co-culture either at the beginning of the culture (time = 0) or after 5 min, 120 min or 24 hrs of initiating the co-cultures. mβCD - 7-KC complexes effectively targets 7-KC to the plasma membrane, but low concentration (0.3 mM) of MβCD does not disrupt lipid rafts [50]. In some experiments 7-KC-mβCD complexes were incubated with either lymph node cells or purified CD4^+^ T cells for 15 minutes and unbound complexes were washed twice by adding excess of wash media and centrifuging the cells. This unique experimental design allowed us to specifically examine effects of 7-KC on lymph node or CD4^+^ T cell membrane at higher concentrations without effects on cellular viability.

### Flow cytometry

Cultured cells were split and one half of cell cultures were labeled with anti-CD4-PE (BioLegend, San Diego, CA) to enumerate proliferating CD4^+^ T cells and the other half was incubated with propidium Iodide (PI) (BD Biosciences, East Rutherford, NJ, USA) to assess the viability of cells as per previously published procedures from our laboratory [49]. Labeled and unlabeled (CFSE/anti-CD4-PE/PI) cells were analyzed by FACS Calibur flow cytometer (BD Biosciences, East Rutherford, NJ, USA) using CellQuestPro software. Moreover, cell cultures set-up with CFSE-labeled lymph node or purified CD4^+^ T cells but left untreated (absence of control and stimulatory c-Ova peptide and 7-KC) were used for setting up the quadrant gate to enumerate non-proliferating cells (upper right quadrant). For assessing the effect of peptide and 7-KC on CFSE-labeled cells, CFSE^highest^ non-proliferating cells present in the upper right quadrant were enumerated as it provides estimation of fraction of cells that did not undergo proliferation as opposed to cells that had undergone symmetrical and non-symmetrical cell division. Statistical significance between the 7-KC treated and untreated groups was determined by one-way analysis of variance tests using JMP software program. The Bonferroni procedure was then completed to determine which means were significantly different from the untreated groups.

### FRET

To assess proximity of two proteins present within the lipid raft on the plasma membrane we carried out FRET analyses. Cells were labeled with a combination of acceptor antibody-conjugated fluorophore, anti-Thy-1-Alexa647 (FL3) (BD Biosciences, Franklin Lakes, NJ, USA), either in combination with donor antibody-conjugated fluorophore, anti-CD3ε mAb-PE (FL2) (eBiosciences, San Diego, CA, USA) or anti CD71-PE (FL2) (BD Biosciences, Franklin Lakes, NJ, USA) for 60 minutes on ice. Cells were washed twice with 1 ml of ice cold 0.1 M PBS and re-suspended in 250 μl of ice cold PBS for FRET analysis with a FACSCalibur flow cytometer (BD Biosciences, Palo Alto, CA) as per previously published procedures [51] with modifications described here. Donor fluorophore (anti-CD3ε-PE) was excited by 488 nm argon laser. Donor emission was detected in FL2 channel (575BP). Compensations were set up with donor-only labeled cells resulting in absence of detection for the donor emission in FL3 channel (620BP). Acceptor-only labeled cells were used to set up background and compensation for FL3 channel resulting in absence of observable emission detected in this channel when excited by 488 nm laser. Emission from the donor and acceptor fluorophore double labeled cells was used to enumerate FRET- positive events, where the emission from the donor (PE) when excited by 488 nm in turn excited the acceptor (AlexaFluor 647). The emission from the acceptor fluorphore was detected in FL3 channel. Cells left unlabeled were used to set-up the negative control gates of the quadrant to assess FRET positive events.

### Staining with Di-4-ANEPPDHQ

Membranes of isolated lymph node cells were stained with di-4-ANEPPDHQ (Invitrogen-Life Technologies, Grand Island, NY) at 0.5 μM final concentration for 20 min at room temperature as suggested by the vendor and previously published protocols [52]. Cells were co-stained with anti-CD90-AlexaFluor 647 and analyzed by FACSCalibur flowcytometer (BD Biosciences, East Rutherford, NJ, USA) using 488 nm and 630 nm (Red) excitation lasers. Emission from these flourophores was measured at wavelengths 570 (FL2 channel), 630 (FL3 channel) and 670 (FL4 channel) after appropriate compensation set-up to allow the detection of specific flourophore. To specifically assess the membrane order of T cells, Anti-CD90-AlexaFluor 647^+^ cells were gated for their assessment of Di-4-ANEPPDHQ staining (FL2 Vs FL3).

## Results

### 7-KC disrupts membrane rafts reversibly

**Figure 1:**
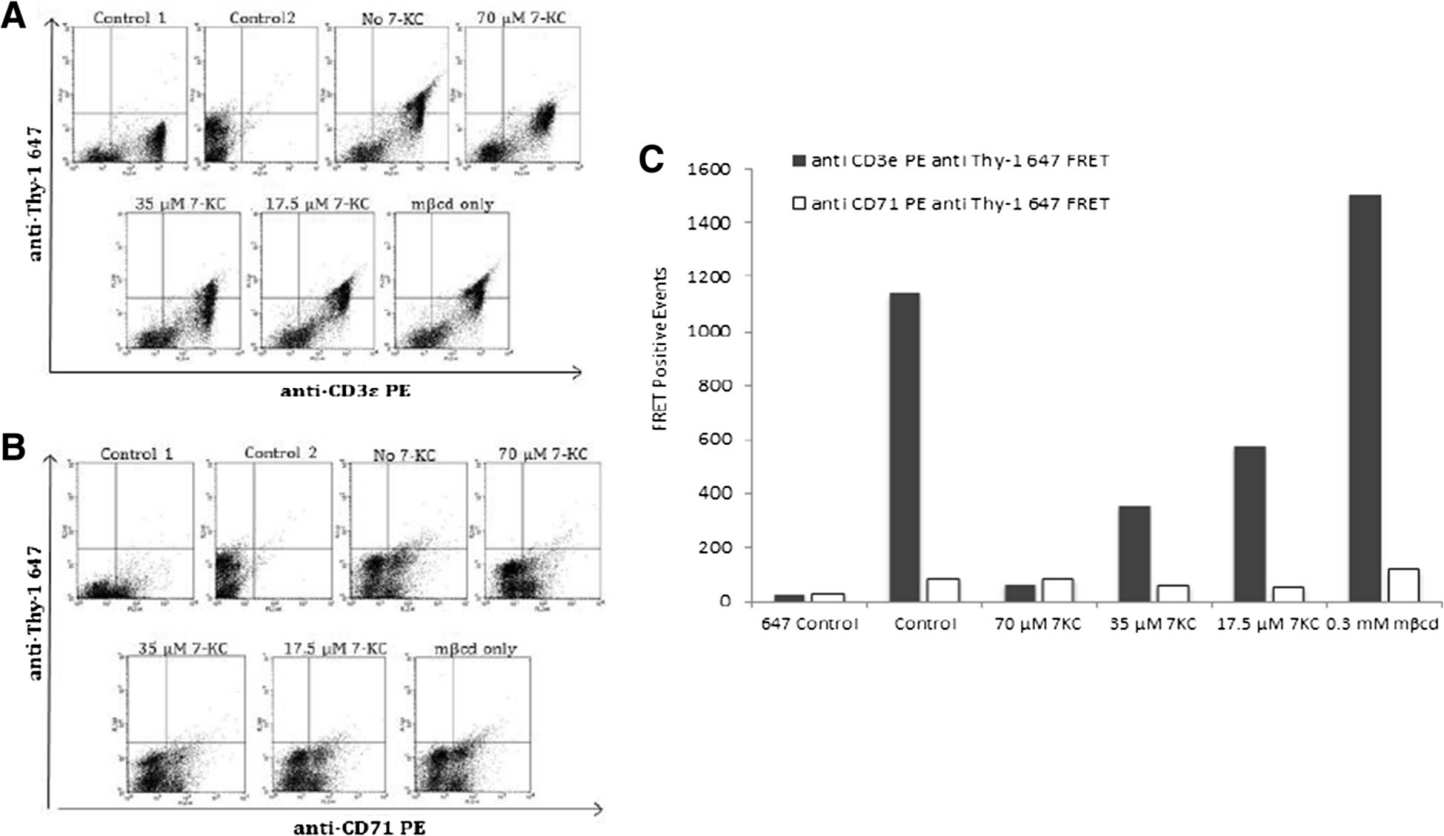
**Exposure to 7-KC inhibits FRET between CD3**ε **and Thy-1 molecules expressed on plasma membrane of CD4** ^**+**^ **T cells.** FRET between Thy-1 and CD3ε was established using donor and acceptor pair of anti-CD3ε PE (FL2) and anti-Thy1 AlexaFluor 647 (FL3) in lymph node cells respectively. **(A)** To assess the FRET positive events the quadrant gates were set up in the absence of either the acceptor (control 1) or donor (control 2). Upper right quadrant shows FRET positive events when cells were stained with both the donor (anti-CD3ε) and acceptor (anti-Thy-1) fluorophore-conjugated antibodies with or without exposure to three different concentrations of 7-KC. **(B)** Representative images of control FRET experiment using anti-CD71 PE (FL2) as FRET donor and Anti-Thy1 AlexaFluor 647 (FL3) as acceptor. **(C)** Graphic representation of the number of events observed in **A** & **B**. A representative experiment of three independent replicates is shown.

To begin to investigate the role of membrane order in clonal expansion of CD4^+^ T cells we first sought to examine whether 7-KC did indeed disrupt the integrity of lipid rafts on the plasma membrane. To accomplish this, we designed an antibody FRET experiment for which we used a FRET pair of an AlexaFluor 647-conjugated antibody against the membrane raft marker Thy1 (acceptor) and a PE conjugated antibody against CD3ε, a component of TCR/CD3 signaling complex (donor). While Thy-1 is known to reside in lipid rafts, the TCRαβ/CD3 complex is also present in these nanodomains [17,42]. In the absence of either the donor (anti-CD3ε PE – FL2 channel) or the acceptor (anti-Thy-1-AlexaFluor647 – FL3 channel) we did not observe cells with productive FRET signal (upper right quadrant) (Figure 1A, controls 1 & 2) by flow cytometer. These negative control experiments were used to set up the gates to enumerate cells with productive FRET signal (events in the upper right quadrant of the two color histogram). To examine the productive FRET signal we stained primary CD4^+^ T cells with anti-CD3ε PE and anti-Thy1-AlexaFluor-647. (Figure 1, no 7-KC group). Productive FRET signal in these cells is indicated by detection of emission from AlexaFluor647 (cells in upper right quadrant). Exposure of CD4^+^ T cells to 7-KC diminished the number of cells with productive FRET signal. Inhibition of FRET signals by incorporating 7-KC in the membrane was dependent on the dose of 7-KC, with a strong FRET quenching observed at 70 μM of 7-KC (>90% inhibition) and low (~50% quenching observed with 17.5 μM 7-KC (Figure 1A and C). To examine the specificity of inhibition by 7-KC we performed a control FRET experiments with anti-CD71 (anti-transferrin receptor), a non-raft marker. CD71 protein is homogenously distributed along the plasma membrane, but excluded from lipid rafts [53]. As expected, we did not observe significant alterations in number of cells emitting FRET signals between CD71-PE and Thy1-AlexaFluor647 in either the presence or absence of 7-KC (Figure 1B and C) at all the 7-KC concentrations tested. Quantitatively the FRET signal was similar to the background FRET with either the donor only (anti-CD3ε-PE) or acceptor (Thy-1 AlexaFluor647) only fluorophore (Figure 1, control 1 and Control 2 groups). These control experiments were also used to establish appropriate gates to set up the thresholds to quantify FRET positive cells (Figure 1A).

**Figure 2:**
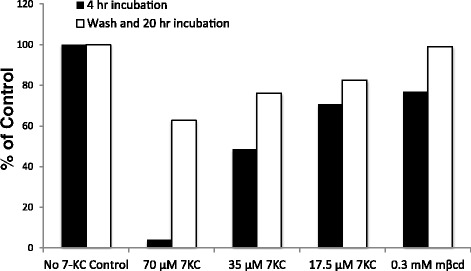
**Reversibility of effects of 7-KC on abolishing FRET between Thy-1 and CD3ε present on membrane rafts on CD4** ^**+**^ **T cells from the lymph node.** FRET between Thy-1 and CD3ε molecules was established using a donor and acceptor pair of anti-CD3ε PE and anti-Thy1 AlexaFluor 647 respectively after CD4^+^ cells were treated with a range of 7-KC concentrations for 4 hrs or left untreated. Reversibility of FRET was assessed after washing excess 7-KC and re-culturing cells for an additional 20 hrs. A representative experiment of three independent replicates is shown.

We next sought to examine if this disruption of lipid raft-based membrane order was reversible. To test that we carried out antibody FRET on CD4^+^ T cells previously exposed to 7-KC after their culture under cholesterol-rich culture conditions (culture media with 10% FBS) for 20 hrs. The FRET signal was restored in cells re-exposed to cholesterol during the 20 hour incubation period (Figure 2). CD4^+^ T cells that were not exposed to 7-KC and cultured under similar culture conditions served as controls and showed a strong FRET signal (Figure 2). These data indicate that the effects of 7-KC on the plasma membrane are reversible with almost complete restoration of the membrane order after 20 hours and therefore suggesting the dynamic nature of these nanodomains. Taken together, these data suggest that the addition of 7-KC to the T cell membrane specifically prevents interaction between the two raft molecules, Thy1 and CD3ε in a reversible way.

**Figure 3:**
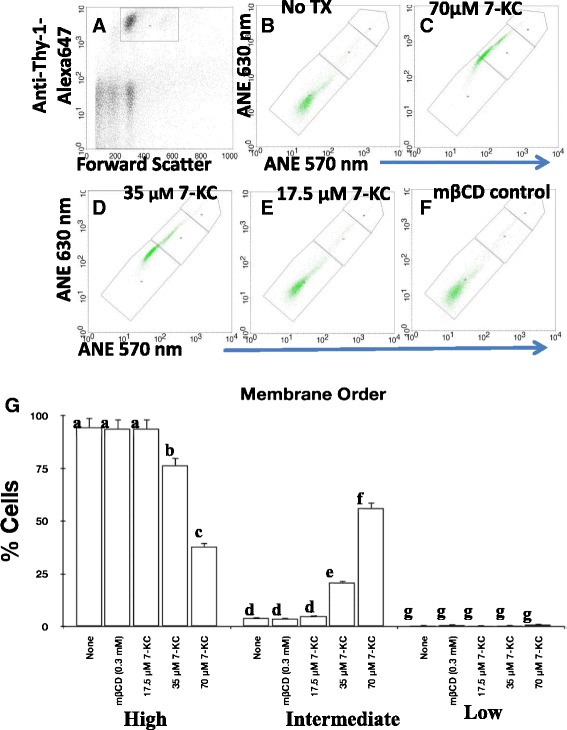
**Exposure to 7-KC alters membrane order in CD4** ^**+**^ **T cells.** Lymph node cells were exposed to di-4 ANEPPDHQ dye and stained with anti-Thy-1 AlexaFluor 647. T cells treated with 70 μM **(C)**, 35 μM **(D)**, 17.5 μM **(E)**, mβCD-vehicle control **(F)** or left untreated **(B)** were gated **(A)** for Thy-1^+^ (FL4) and assessed for emission at 570 nm (FL2 channel) and 630 nm (FL3 channel) after appropriate compensation. T cells with high (lower most gate in B-F panels), intermediate (middle gate in B-F panels) and low (higher most gate in B-F panels) membrane order were enumerated and graphic representation of percent cells from four independent experiments with high, intermediate and low membrane order is shown in **(G)**. Statistical significance between untreated, 7-KC treated groups was computed by two way ANOVA using JMP program. Different lower case alphabet designations (a – g) above each treatment group (none/untreated, mβCD, 17.5 μM, 35 μM, 70 μM 7KC) indicates statistically significant difference (p <0.0001) within the group of cells with high or intermediate or low membrane order. Similar lower case alphabet designation indicates lack of statistical significance (p > 0.8371).

To assess alterations in membrane fluidity by 7-KC we chose di-4-ANEPPDHQ dye, this dye senses the membrane order and reports it on FL2 (570 nm) and FL3 (630 nm) channels when excited by 488 nm laser [52,54]. Figure 2 shows that gated (Thy-1^+^) lymph node T cells (Figure 3A) showed altered membrane order after their exposure to 7-KC in a concentration dependent manner (Figure 3C-E) when compared to the untreated cell controls (Figure 3B). Alterations of membrane order by 7-KC were significant, the highest effect was observed at two higher concentrations of 7-KC (70 μM & 35 μM) (Figure 3B-E & G). The observed effect of 7-KC was specific as the cells treated with mβCD vehicle control (Figure 3F & G) did not show alterations in membrane order when compared to cells that were left untreated (Figure 3B & G). Taken together these data are consistent with the FRET data and suggest that CD4^+^ T cell membranes exposed to 7-KC alter membrane order.

### T cell activation and proliferation is disrupted by 7-KC without altering viability of these cells

**Figure 4:**
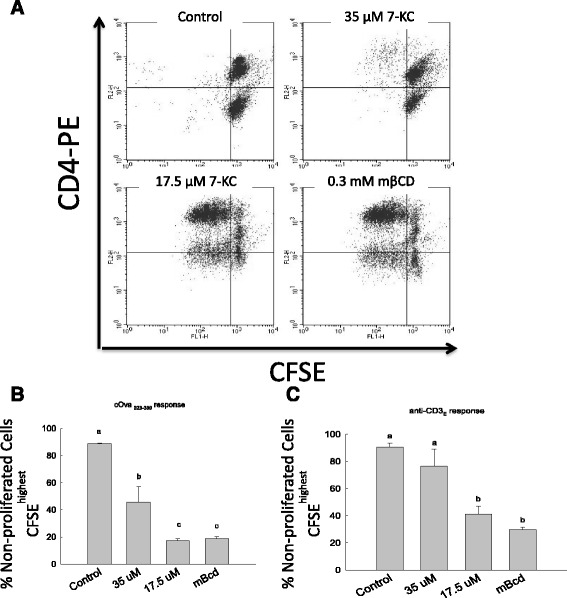
**Inhibition of antigen-specific proliferation of CD4** ^**+**^ **T cells by 7-Keto cholesterol in dose-dependent fashion. A**. Lymph node cells were cultured with c-Ova_323–339_ stimulatory or c-Ova_324–334_ control peptide **(A & B)** or anti-CD3ε monoclonal antibody **(C)** either in the absence or presence of 7-KC. Non-dividing cells were enumerated by two color staining with CFSE (X-axis) and anti-CD4-PE (Y-axis), a representative experiment is shown **(A)**. Responsiveness to stimulation through the antigen receptor was quantified by enumerating non-proliferating cells (upper right quadrant) in these cultures **(B & C)**. These data were obtained from three independent experiments. Statistical significance between treated and untreated groups was computed by one way ANOVA using JMP program. Different lower case alphabet designations (a, b, c) above each treatment group (control, 35 μM 7KC, 17.5 μM 7KC, mβCD) indicates statistically significant difference (p <0.001) in the presence of either cOva_323–339_ peptide or anti-CD3ε antibody. To examine statistical significance within a specific antigen receptor response (cOva_323–339_ and anti-CD3ε), each treatment group (e.g., 7KC) is compared with other (all possible combinations). Similar lower case alphabet designation indicates lack of statistical significance (p > 0.05).

To examine the role of lipid raft-based membrane order in antigen-specific clonal expansion, lymph node cells from DO11 TCR transgenic mice were first labeled with CFSE followed by incubation with different concentrations of 7-KC along with either a stimulatory peptide c-OVA_323–339_, a control c-OVA _324–334_ peptide or stimulatory anti-CD3ε monoclonal antibody. Proliferation of CD4^+^ T cells in the culture was examined by FACS after staining with anti-CD4 antibody to enumerate CD4^+^ T cells. Day-3 cultures also were stained with propidium iodide to assess viability of cells in these cultures. Whole range of concentrations for 7-KC was assessed for its effect on cell viability (data not shown). Based on these initial experiments 35 μM and lower concentrations of 7-KC was used for the experiments here. Figure 4A shows that 35 μM 7-KC showed a significant inhibition of CD4^+^ T cell proliferation induced by c-OVA_323–339_ (Figure 4A and B) and anti-CD3ε (Figure 4C). In the presence of non-stimulatory peptide cOVA_324–334_ (Figure 4A and B) or anti-CD3ε (Figure 4C) about 90% cells expressed non-dividing phenotype (CD4^+^CFSE^highest^ cells in upper right quadrant), as expected. In contrast, as many as 45% or 76% of cells had not undergone cell division (CD4^+^CFSE^highest^) in the presence of specific stimulatory peptide or anti-CD3ε mAb respectively. Minimal inhibition was observed in the presence of 17.5 μM 7-KC as only 17.3% and 41.2% non-dividing CD4^+^CFSE^highest^ T cells remained in the cultures stimulated with cOva_323–339_ and anti-CD3ε respectively (Figure 4A and B). In contrast, we observed clonal expansion of untreated CD4^+^ T cells and CD4^+^ T cells treated with vehicle control (mβCD) only in the presence of cOva_323–339_ (Figure 4A and B) or anti-CD3ε antibody (Figure 4C), as expected. Only 21% (for cOva_323–339_ stimulated cultures) and 31% (for anti-CD3ε stimulated cultures) of day-3 CD4^+^ T cells had not undergone cell division. To examine the downstream effects of altered membrane order we next examined the effects of 7-KC on cytokine production. CD4^+^ T cells exposed to 7-KC showed reduced IFN-γ production and these effects were directly proportional to the concentration of 7-KC treatment over the three day period (Additional file 1: Figure S1). Highest inhibition was observed in cultures with 70 μM and 35 μM 7-KC. These inhibitory effects were specific as the cytokines produce in cultures with vehicle control, mβCD, generated similar levels of cytokines as cell that were not treated with 7-KC.

**Figure 5:**
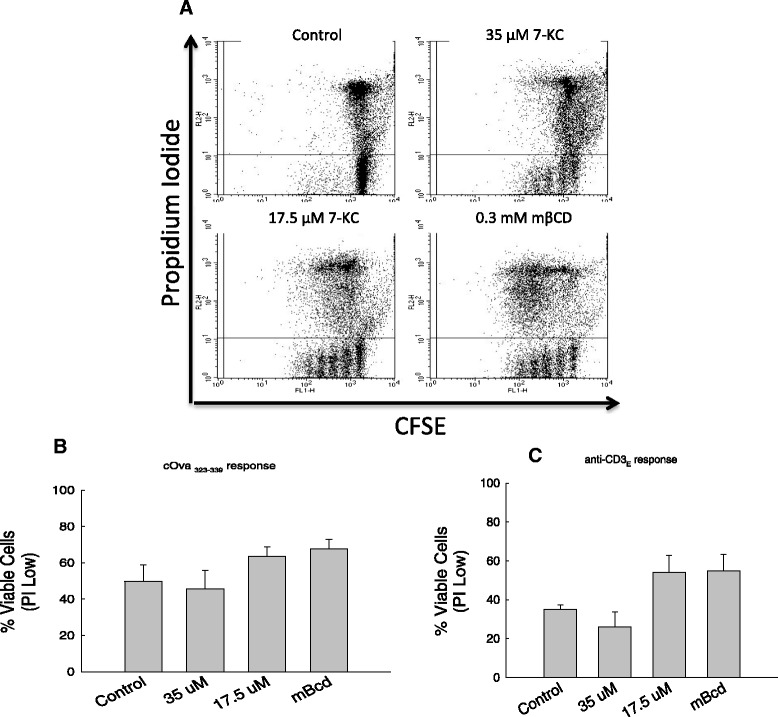
**Assessment of viability of CD4** ^**+**^ **T cells treated with 7-Keto cholesterol. A**. Lymph node cells were cultured with 35 μM, 17.5 μM 7-KC, mβCB vehicle control either in the presence of c-Ova_323–339_ peptide (Panel **B**) or anti-CD3ε monoclonal antibody **(C)**. Viable cells were enumerated by staining with CFSE (X-axis) and Propidium Iodide (Y-axis). Live cells in these cell cultures were quantified by enumerating CFSE^high^ PI^negative^ cells. These data graphically represented in panels **B** & **C** are obtained from three independent experiments. A representative flow analysis of one of the three experiments computed for panel C is shown in panel A. Statistical significance between treated and untreated groups was computed by one way ANOVA using the JMP program. Similar lower case alphabet designations (a) above each treatment group (control, 35 μM 7KC, 17.5 μM 7KC, mβCD) indicates lack of statistically significant differences (p = 0.21 for cOva peptide responses and p = 0.06 for anti-CD3ε responses). To examine statistical significance within a specific antigen receptor response (cOva_323–339_ or anti-CD3ε), each treatment group (e.g., 7KC) is compared with other (all possible combinations).

To examine the specificity of 7-KC treatment on clonal expansion of CD4^+^ T cells we assessed the viability of day-3 cell cultures. Figure 5 shows that cells treated with lower 7-KC concentrations (35 & 17.5 μM) did not affect cell viability (Figure 5A-B) compared to cells treated with cOVA_324–334_ control peptide alone or treated with vehicle control (mβCD), but the clonal expansion of CD4^+^ T cells in response to c-OVA_323–339_ peptide and anti-CD3ε monoclonal antibody was significantly reduced in the presence of 35 μM 7-KC (Figure 4). Reduced viability of normal CD4^+^ T cells in controls on prolonged cell culture is well known and previously documented by us as well [49]. Taken together these data suggest that disruption of lipid raft-based membrane order with insertion of 7-KC in the membrane inhibits antigen receptor-driven clonal expansion of CD4^+^ T cells and cytokine production without compromising the viability of the cells.

### Role of lipid raft-based membrane order in the temporal sequence of CD4^+^ T cell clonal expansion

**Figure 6:**
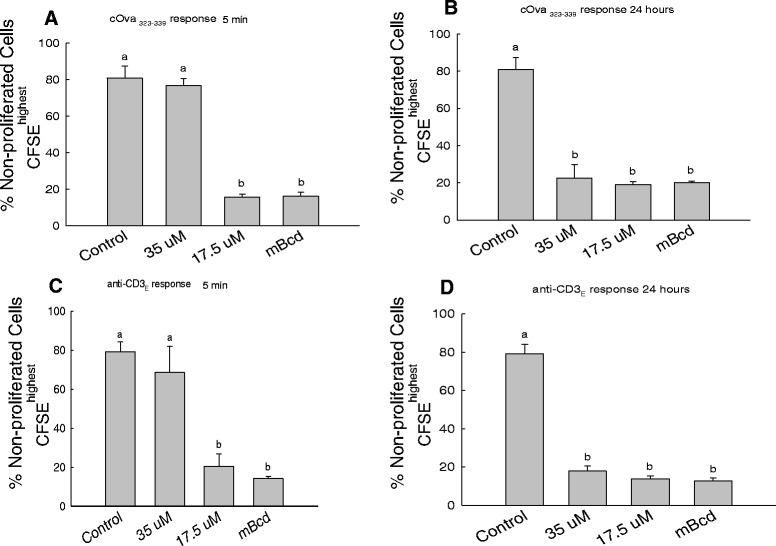
**Inhibitory effects of 7-Keto cholesterol on antigen-specific CD4** ^**+**^ **T cell proliferation occur within first two hours of antigen receptor engagement.** Lymph node cells were cultured with c-Ova_323–339_ peptide **(A & B)** or anti-CD3ε monoclonal antibody **(C & D)**. 7-KC was added to the cultures either at 5 min **(A & C)** or at 24 hrs **(B & D)** after establishing cultures. Proliferating and non-proliferating cells were enumerated by two color staining with CFSE and anti-CD4-PE, and graphical representation of non-proliferating cells (CFSE^highest^) CD4^+^ T cells in cultures is shown. These data were obtained from three independent experiments and for each time point was collected in triplicate. Statistical significance between treated and untreated groups was computed by one way ANOVA using the JMP program. Different lower case alphabet designations (a, b) above each treatment group (control, 35 μM 7KC, 17.5 μM 7KC, mβCD) indicates statistically significant difference (p <0.0001) with in each timed addition of 7KC and agonist used for T cell response (cOva_323-339_ 5 minute/24 hr or anti-CD3ε 5 minute/24 hr). To examine statistical significance within a specific antigen receptor response (cOva_323–339_ or anti-CD3ε), each treatment group (e.g., 7KC) is compared with other (all possible combinations). Similar lower case alphabet designation indicates lack of statistical significance (p > 0.05).

We next sought to test the time at which 7-KC exerted its effect on CD4^+^ T cell clonal expansion in response to a specific antigen. The early events within seconds and minutes of engagement of the antigen receptor are associated with activation of membrane kinases and phosphorylation events as well as generation of secondary messengers. The later events (after gene transcription) are associated with mitosis primarily driven by the growth factor IL-2. To examine whether the presence of 7-KC affected clonal expansion by altering lipid raft-based membrane order at an early or later phase of clonal expansion, we added this compound at different time points after engaging the antigen receptor. Figure 6A and C & Additional file 2: Figure S2 show that addition of 7-KC at 5 minute time point after the cultures were set up. Addition of 7-KC at these time points dramatically inhibited the clonal expansion of CD4^+^ T cells. In the presence of 35 μM 7-KC (a concentration at which the viability of CD4^+^ T cells is not affected) we observed significant reduction in clonal expansion of CD4^+^ T cells in response to stimulation through the antigen receptor. Quantifying day-3 cell cultures that were set-up in the presence of 35 μM 7-KC indicated that about 80% and 70% of CD4^+^ cells remained unresponsive (not undergone cell division) to c-OVA_323–339_ peptide and anti-CD3ε, respectively (Figure 6A and C & Additional file 2: Figure S2A & B) which was similar to the control cultures set up with control cOVA_324–334_ peptide (Figure 6A & Additional file 2: Figure S2A) or left untreated (Figure 6C & Additional file 2: Figure S2B) showing approximately 80% CD4^+^CFSE^highest^ non-proliferating cells. Proportionally lower inhibition was observed with 17.5 μM 7-KC with about 15% and 20.5% of non-divided cells remaining in the cultures with cOva_323–339_ peptide and anti-CD3ε antibody stimulated cells respectively. Fraction of non-proliferating cells in the cultures was not significantly different from the cultures stimulated with cOVA_323–339_ in the presence (Figure 6A and C & Additional file 2: Figure S2) or absence (data not shown) of vehicle mβCD. Similar results were obtained when 7-KC was added at 2 hours after initiating the co-cultures (Additional file 2: Figure S2). In contrast, addition of 7-KC at 24 hrs time point after the engagement of antigen receptor did not affect clonal expansion of CD4^+^ T cells (Figure 6B and D & Additional file 2: Figure S2). These results suggest that intactness of membrane order is critical during early signaling events. Taken together, these data suggest that lipid raft-based membrane order contributes to clonal expansion during the early phase (occurring within 2 hrs.) of the response triggered through engagement of the antigen receptor.

### Disrupting lipid raft-based membrane order by incorporating 7-KC in CD4^+^ T cells is sufficient to inhibit their clonal expansion in response to cOVA_323–339_

**Figure 7:**
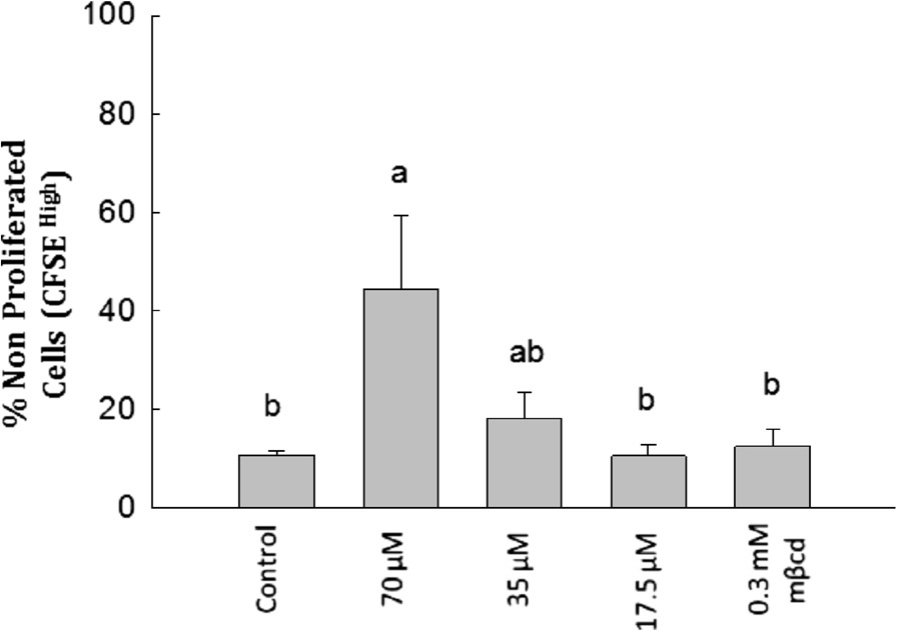
**Effect of 7-KC on the proliferation of CD4** ^**+**^ **T cell and APC co-cultures where only CD4** ^**+**^ **T cells were pre-treated with 7-KC.** Purified CD4^+^ T cells were incubated with 7-KC for 30 minutes at 37°C and excess of 7-KC was removed by washing with wash media. CD4^+^ T cells were stained with CFSE prior to being combined with APCs. 7-KC treated and mock-treated (labeled as control) CD4^+^ T cells were cultured with c-Ova_323–339_ peptide for 72 hrs and stained with anti-CD4^+^ PE enumeration of CD4^+^ T cells only as described in materials and methods section. Proliferating and non-proliferating cells were enumerated by two color staining with CFSE (X-axis) and anti-CD4-PE (Y-axis) and graphical representation of non-proliferating cells (CFSE^highest^) CD4^+^ T cells in cultures is shown. These data were obtained from three independent experiments and each 7-KC concentration was carried out in triplicates. Statistical significance between treated and untreated groups was computed by one way ANOVA using JMP program. Different small alphabet designations above each bar indicate statistically significant differences (p < 0.0221). Different lower case alphabet designations (a, b, c) above each treatment group (control, 35 μM 7KC, 17.5 μM 7KC, mβCD) indicates statistically significant difference (p < 0.0221) in response to either cOva_323–339_ peptide or anti-CD3ε antibody. To examine statistical significance within a specific antigen receptor response (cOva_323–339_ or anti-CD3ε), each treatment group (e.g., 7KC) is compared with other (all possible combinations). Similar lower case alphabet designation indicates lack of statistical significance (p > 0.05). Double designation (ab) indicates lack of statistical significance with experimental treatments with designations “a” as well as “b”.

Next we sought to examine if lipid raft-based order specifically on the plasma membrane CD4^+^ T cells contributed to diminished clonal expansion of these cells in response to antigen. To test this we incorporated 7-KC in the membrane of purified CD4^+^ T cells for brief period (excess of 7-KC was removed) and tested their clonal expansion in response to c-OVA_323–339_ presented by APCs that were not exposed to 7-KC. Brief treatment (15 min) with 7-KC allowed us to examine the effects of 7-KC at higher concentration (70 μM). Figure 7 shows that CD4^+^ T cells with 7-KC incorporated in the plasma membrane showed reduced clonal expansion in a dose-dependent fashion. A maximum effect was observed with 70 μM of 7-KC and minimal inhibition. As much as, 44.2% of CD4^+^ T cells incubated with 70 μM 7-KC and 18% of CD4^+^ T cells incubated with 35 μM 7-KC did not proliferate in response to c-OVA_323–339_ peptide. In the absence of 7-KC and in the presence of vehicle control mβCD, only 10.6% and 15% of CD4^+^ cells showed lack of proliferation in the OVA_323–339_ stimulated cultures (Figure 7). These data indicate that disturbance of membrane order on CD4^+^ T cell membrane contributes to their diminished clonal expansion in response to the antigen receptor.

## Discussion

Asymmetrical distribution of lipids with in the membrane bilayer and their organization in association with or without membrane proteins into nano-domains (lipid rafts) are the two fundamental properties of the plasma membrane. Lipid raft nano-structures enriched in cholesterol, saturated lipids, and lipid-modified signaling proteins that assemble into dynamic, compositionally heterogeneous nano-domains similar to the liquid order (I_o_) phase observed with model membranes [55]. These assemblies of proteins and lipids, because of the heterogeneity in composition compartmentalize signaling and, contribute to spatial asymmetry of the membrane bilayer, as well. One approach used to assess the role of lipid raft-based order in cell signaling relies on disrupting the membrane order by exposing the cell membrane to cholesterol-binding, raft-destabilizing molecule, MβCD. A considerable number of published studies where the role of lipid rafts in early signaling events was assessed have used this compound [43,44]. However, concerns related to its effectiveness at high concentrations and its adverse effects on internal Ca^2+^ stores have remained [45]. More recently, 7-Keto cholesterol was shown to interfere with the lipid raft-based membrane order in immortalized cells [41,50,56]. Incorporation of 7-KC resulted in de-condensing of the plasma membrane at the immunological synapse at immortalized CD4^+^ T cell line - APC interface [50]. These findings using immortalized T cell line are consistent with early observations related to the effectiveness of 7-KC in preventing the formation of L_o_ domains in a model membrane [57,58]. However, the role of lipid raft-based membrane order in clonal expansion of primary CD4^+^ T cells has not been examined. Moreover, the role of foreign antigen in driving this clonal expansion is unknown. We have used CD4^+^ T cells from c-OVA_323–339_-peptide specific T cell receptor transgenic mice to examine this question. We observed that 7-KC inhibited CD4^+^ T cell proliferation and IFN-γ cytokine production in response to c-OVA_323–339_ peptide in a concentration dependent manner.

Direct insertion of 7-KC in the membrane can alter raft-based membrane order by destabilizing the membrane ordered phase. The presence of the carbon 7 ketone group on 7-KC prevents the tight packing of saturated acyl chains needed for the formation of the L_o_ phase as observed in model membranes. Consistent with this idea was the observation that 7-KC altered membrane order when assessed with di-4 ANEPPDHQ. In addition, the antibody FRET experiments we observed a significant decrease in FRET signal between CD3ε and Thy-1 in the plasma membrane of cells treated with 7-KC indicated an altered membrane fluidity. It is also likely that insertion of 7-KC in the membrane breaks spatial asymmetry and compartmentalization of signaling receptors that negatively impacts early membrane proximal cell signaling events and in turn on the clonal expansion of CD4^+^ T cells in response to a specific antigen.

Antigen-driven clonal expansion of CD4^+^ T cells can be viewed to occur in two major phases. In the first phase peptide and MHC class II recognition by the antigen receptor, along with the interaction of a plethora of accessory cell proteins, results in CD4^+^ T cell activation. The activated CD4^+^ T cells generate IL-2, a growth factor for T cells. In the second phase the IL-2 driven clonal expansion occurs. Previous reports investigating the role of lipid rafts using MβCD in T cell signaling had suggested that disruption of lipid rafts interferes in the association of a number of key kinases to the lipid raft [43,44]. Our timing experiments where clonal expansion of CD4^+^ T cell is the final read-out suggests that disruption of membrane order exerts its effect during the CD4^+^ T cell activation phase, as significant alteration in clonal expansion of these cells was not observed when 7-KC was added to the cell cultures 24 hrs after engaging the antigen receptor. Our data with primary CD4^+^ T lymphocytes shows that 7-KC possibly alters the association or stability of signaling complexes during early phases of T cell activation. Alternatively, destabilizing lipid rafts by incorporating 7-KC can result in the degradation of Akt and/or disruption of ras nanodomains present in the inner leaflet of the plasma membrane [59-62]. Overall our results are consistent with the observation that subsets of primary CD4^+^ T cells present in peripheral human blood show functional responses correlating with membrane lipid order. The highest response generated by CD4^+^ T cells through its TCR was observed with high lipid order and lower responses correlated to cells with low membrane lipid order [52].

Cholesterol has the ability to create order in the lipid bilayer of the plasma membrane in variety of cell types [63]. The rigid structure of the cholesterol molecule allows for tight organization of the bilayer. Above a certain cholesterol threshold level, cholesterol rich (or L_o_) and cholesterol poor (L_d_) phases can exist in a membrane, while below this threshold only L_d_ phases are observed. The presence of cholesterol prevents the deformation of the lipid acyl chains and allows for the movement of small molecules across the bilayer, while lipids can still move freely past each other. Several studies have confirmed these findings by demonstrating that certain levels of cholesterol induce a phase transition. Theoretically, cholesterol levels could be increased to the level where the entire membrane is a lipid ordered phase. A study (independent of the lipid raft hypothesis) by Bensinger et al. in 2008 has demonstrated that cholesterol is critical for T cell proliferation [64]. Their data showed that disrupting LXR genes, which are involved in the transcriptional regulation of intracellular cholesterol homeostasis, caused a loss of control of the immune response and hyperplasia in affected T cells. These experiments highlight the importance of cholesterol homoeostasis in cell signaling. Consistent with this is the data that by activating LXR results in reduced levels of membrane cholesterol affecting the membrane order [65]. These published data suggest that homoeostasis of cholesterol regulates proliferative potential of T cells possible by altering cholesterol-dependent membrane order.

One mechanism through which lipid raft-based membrane order may contribute to cell signaling is by promoting proximity between signaling proteins in the membrane, which allows signaling for cell survival in a ligand-independent manner through cell autonomous (external ligand-independent) mechanisms or tonic signaling mechanisms. However, higher order membrane coalescence and compartmentalization of signaling molecules in and out of these aggregated rafts is promoted by specific ligand – receptor interactions in a non-cell autonomous manner. We think that 7-KC disrupts lipid raft-based membrane order and the assembly of lipid rafts during interaction between the interacting CD4^+^ T cells and APCs and thereby inhibiting clonal expansion of CD4^+^ T cells in response to engagement of antigen receptors. Our results also suggest that re-organization of lipid rafts and membrane order has a role in the early part of the T cell response as opposed to the second phase that is driven by IL-2 binding to the IL-2 receptor. This early signaling is likely to inhibit the gene expression patterns as indicated by inhibition of IFN-γ cytokine production by 7-KC- exposed CD4^+^ T cells in response to the antigen receptor signaling. Further experiments will be required to directly identify the molecular players at the early stages of CD4^+^ T cell activation influenced by lipid raft-based membrane order.

## Conclusion

Our findings suggest that lipid raft-based membrane order is critical for clonal expansion of CD4^+^ T cells in response to a model peptide.

## Additional files

Additional file 1: Figure S1. 7-Keto cholesterol inhibits production of IFN-γ by CD4^+^ T cells in response to a specific antigen. Lymph node cells were cultured with c-Ova_323–339_ peptide either in the absence or presence of various concentration of 7-KC. Cells exposed to 0.3 mM mβcd, (vehicle control) (open inverted triangle), 17.5 μM 7-KC (filled diamond), 35 μM (open triangle), 70 μM (open circle) or left untreated (open square) were stimulated with c-Ova_323–339_ and supernatant was harvested on days 1, 2 and 3 to quantify IFN-γ by ELISA (BD Biosciences). Supernatants from cultures stimulated with control peptide, cOva_324–334_ was used as controls (open diamond). A representative data out of total three experiments is shown. Additional file 2: Figure S2. Inhibitory effects of 7-Keto cholesterol on antigen-specific CD4^+^ T cell proliferation occur within first two hours of antigen receptor engagement. Lymph node cells were labeled with CFSE and cultured with c-Ova_323–339_ peptide (A) or anti-CD3ε monoclonal antibody (B). 7-KC was added to the cultures either at 5 min, 2 hrs or at 24 hrs after establishing cultures as indicated in each panel. Proliferating and non-proliferating cells were enumerated by two color analyses with CFSE (FL1) and anti-CD4-PE (FL2). A representative graphs from three independent experiments and for each time point collected in triplicate is shown and quantification of these data for 5 minute and 24 hr time points is shown in Figure 7. Co-culture groups were treated with cOva324-334 peptide (panel A) or left untreated (B). Majority of CD4^+^ T cells (approximately 80%) remain non-proliferated under these conditions.

## Abbreviations

CD4: Cluster differentiation antigen 4
APC: Antigen presenting cell
7-KC: 7-ketocholesterol
GPI: Glycosylphosphotidyl-inositol
TCR: T cell receptor
Th: T helper cell
I_o_: Lipid ordered phase
I_d_: Lipid disordered phase
