# Supplementary material for: Lipid raft-based membrane order is important for antigen-specific clonal expansion of CD4+ T lymphocytes
Source: BMC Immunol. 2014 Dec 14;15:58. doi: 10.1186/s12865-014-0058-8 (PMC4270042; doi:10.1186/s12865-014-0058-8)
Supplement: Additional file 1: Figure S1. — 7-Keto cholesterol inhibits production of IFN-γ by CD4+ T cells in response to a specific antigen. Lymph node cells were cultured with c-Ova323–339 peptide either in the absence or presence of various concentration of 7-KC. Cells exposed to 0.3 mM mβcd, (vehicle control) (open inverted triangle), 17.5 μM 7-KC (filled diamond), 35 μM (open triangle), 70 μM (open circle) or left untreated (open square) were stimulated with c-Ova323–339 and supernatant was harvested on days 1, 2 and 3 to quantify IFN-γ by ELISA (BD Biosciences). Supernatants from cultures stimulated with control peptide, cOva324–334 was used as controls (open diamond). A representative data out of total three experiments is shown. [file 12865_2014_58_MOESM1_ESM.pdf]

Supplementary Figure 1

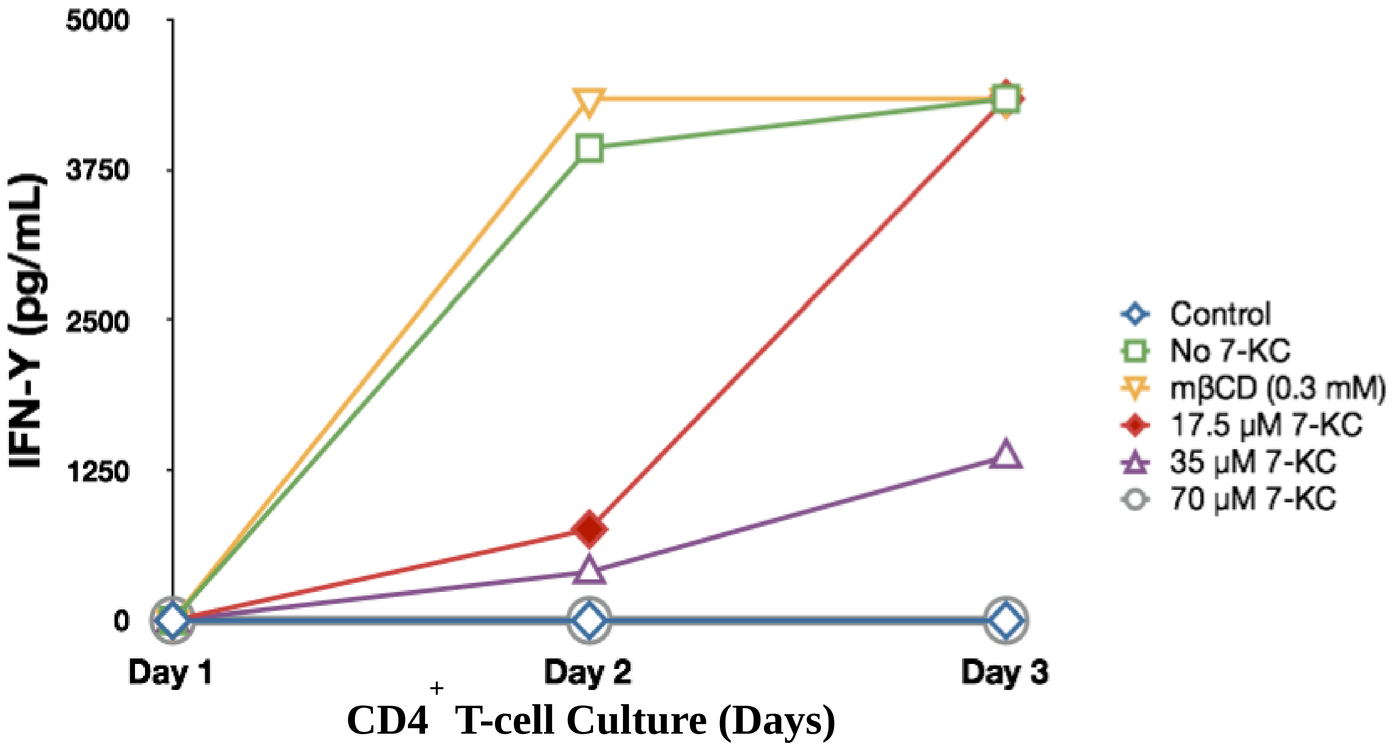

Supplementary Figure 1. 7-Keto cholesterol inhibits production of IFN-γ by $CD4^+$ T cells in response to a specific antigen. Lymph node cells were cultured with c-$Ova_{323\text{-}339}$ peptide either in the absence or presence of various concentration of 7-KC. Cells exposed to 0.3mM mβcd, (vehicle control) (open inverted triangle), 17.5 μM 7-KC (filled diamond), 35 μM (open triangle), 70 μM (open circle) or left untreated (open square) were stimulated with c-$Ova_{323\text{-}339}$ and supernatant was harvested on days 1, 2 and 3 to quantify IFN-γ by ELISA (BD Biosciences). Supernatants from cultures stimulated with control peptide, $cOva_{324\text{-}334}$ was used as controls (open diamond). A representative data out of total three experiments is shown.
