# Supplementary material for: Lipid raft-based membrane order is important for antigen-specific clonal expansion of CD4+ T lymphocytes
Source: BMC Immunol. 2014 Dec 14;15:58. doi: 10.1186/s12865-014-0058-8 (PMC4270042; doi:10.1186/s12865-014-0058-8)
Supplement: Additional file 2: Figure S2. — Inhibitory effects of 7-Keto cholesterol on antigen-specific CD4+ T cell proliferation occur within first two hours of antigen receptor engagement. Lymph node cells were labeled with CFSE and cultured with c-Ova323–339 peptide (A) or anti-CD3ε monoclonal antibody (B). 7-KC was added to the cultures either at 5 min, 2 hrs or at 24 hrs after establishing cultures as indicated in each panel. Proliferating and non-proliferating cells were enumerated by two color analyses with CFSE (FL1) and anti-CD4-PE (FL2). A representative graphs from three independent experiments and for each time point collected in triplicate is shown and quantification of these data for 5 minute and 24 hr time points is shown in Figure 7. Co-culture groups were treated with cOva324-334 peptide (panel A) or left untreated (B). Majority of CD4+ T cells (approximately 80%) remain non-proliferated under these conditions. [file 12865_2014_58_MOESM2_ESM.pdf]

Supplemental Figure 2A

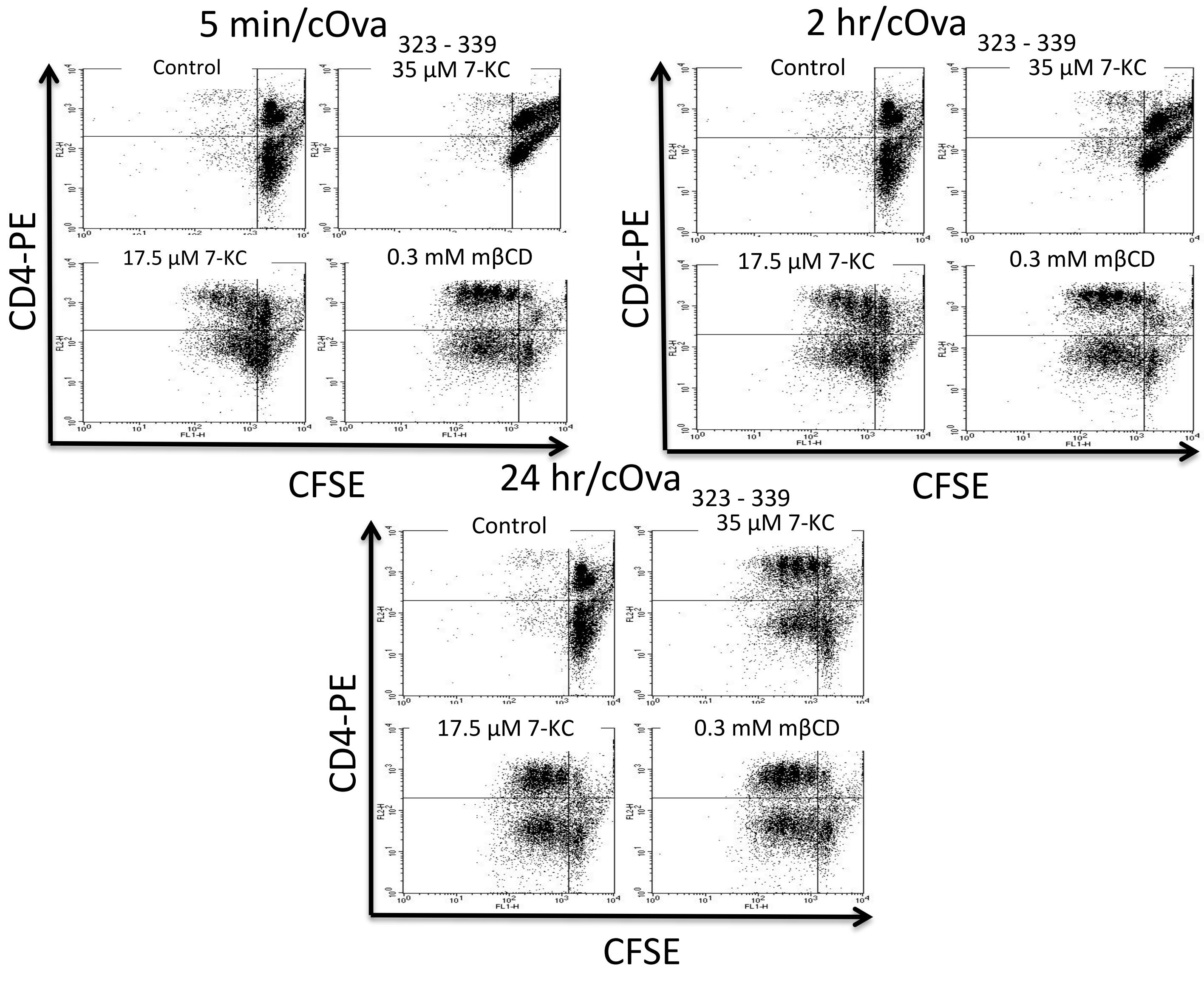

Supplemental Figure 2B

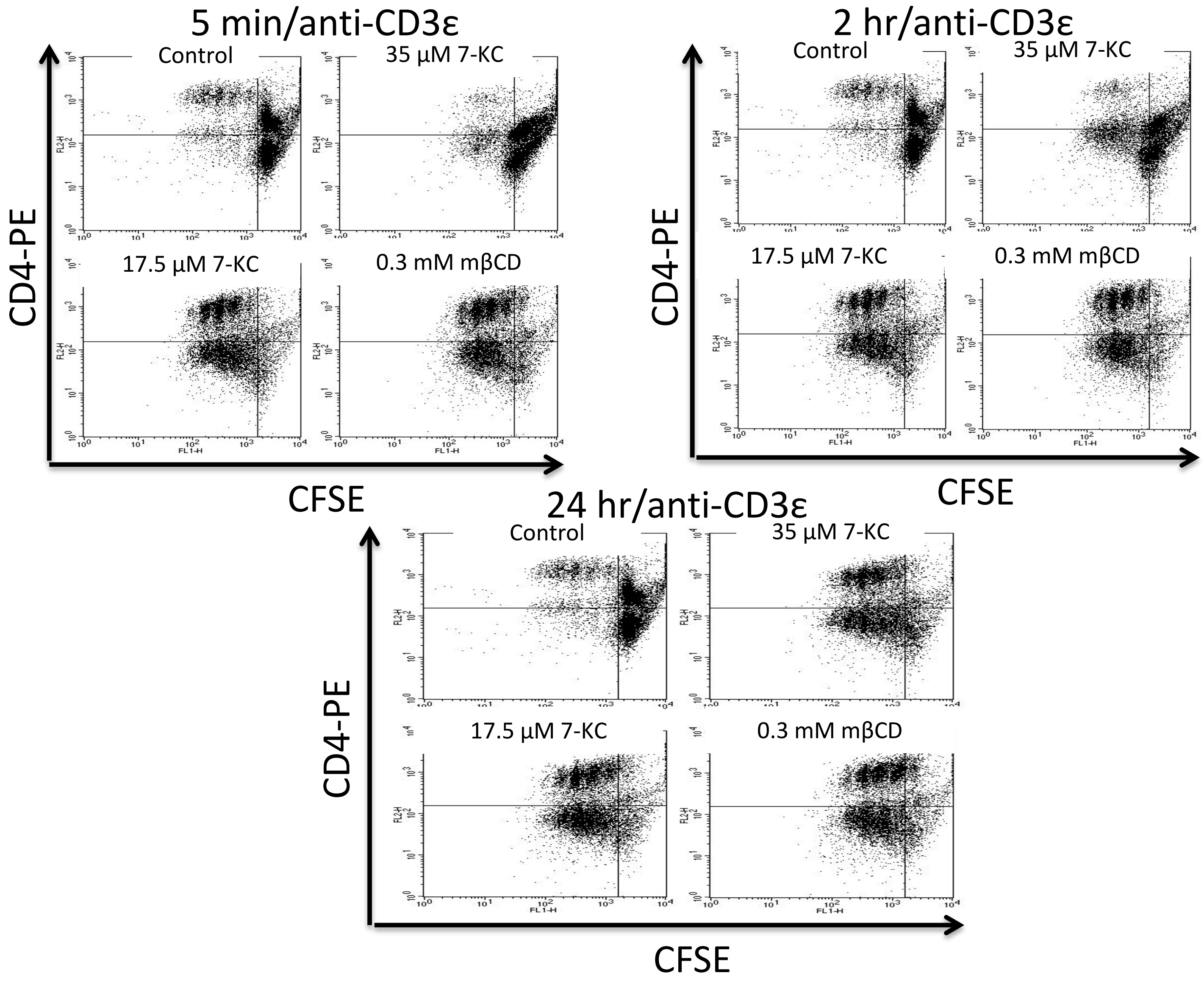

Supplementary Figure 2. Inhibitory effects of 7-Keto cholesterol on antigen-specific $CD4^{+}$ T cell proliferation occur within first two hours of antigen receptor engagement. Lymph node cells were labeled with CFSE and cultured with c-$Ova_{323-339}$ peptide (A) or anti-CD3ε monoclonal antibody (B). 7-KC was added to the cultures either at 5 min, 2 hrs or at 24 hrs after establishing cultures as indicated in each panel. Proliferating and non-proliferating cells were enumerated by two color analyses with CFSE (FL1) and anti-CD4-PE (FL2). A representative graphs from three independent experiments and for each time point collected in triplicate is shown and quantification of these data for 5 minute and 24 hr time points is shown in figure 6.
